# Cytoreductive surgery and hyperthermic intraperitoneal chemotherapy for colorectal peritoneal metastases: a single-institution experience

**DOI:** 10.2478/raon-2026-0041

**Published:** 2026-09-07

**Authors:** Gasper Pilko, Rok Petric

**Affiliations:** 1 Department Surgical Oncology, Institute of Oncology Ljubljana, Ljubljana, Slovenia; 2 Medical Faculty, University of Ljubljana, Ljubljana, Slovenia

**Keywords:** colorectal cancer, peritoneal metastases, cytoreductive surgery, HIPEC, peritoneal cancer index

## Abstract

**Background:**

Peritoneal metastases (PM) from colorectal cancer are associated with poor prognosis. Cytoreductive surgery (CRS) combined with hyperthermic intraperitoneal chemotherapy (HIPEC) has been proposed for selected patients, although its role remains debated. This study evaluates the efficacy and safety of CRS plus HIPEC in a single institution.

**Patients and methods:**

This retrospective study included patients with colorectal PM treated with CRS and HIPEC between 2009 and 2024. Preoperative staging involved CT and/or MRI, and all cases were discussed in a multidisciplinary team. Disease burden was assessed using the Peritoneal Cancer Index (PCI), and completeness of cytoreduction (CC score) was recorded. HIPEC was performed with oxaliplatin (360 mg/m^2^ at 42°C for 90 minutes). Overall survival (OS) was the primary endpoint; disease-free survival (DFS) and morbidity were secondary endpoints.

**Results:**

Fifty patients (mean age 54 years) were included. Complete cytoreduction (CC-0/1) was achieved in 88% of cases, with a median PCI of 8.33. Severe complications occurred in 18% of patients, with no 30-day mortality. After a median follow-up of 34 months, median OS was 49 months; 1- and 5-year OS rates were 96% and 54%, respectively. Median DFS was 14 months. PCI, CC score, and prior surgical score (PSS) were associated with OS on univariate analysis, while PCI and CC score remained independent prognostic factors.

**Conclusions:**

CRS plus HIPEC is a feasible and effective treatment for selected patients with colorectal PM, with acceptable morbidity. Low PCI and complete cytoreduction are key predictors of improved survival.

## Introduction

Colorectal cancer represents the third most common cancer and the fourth most common cause of cancer-related death worldwide.^1^ The peritoneum is the second most frequent site of colon cancer metastasis.^2^ Five percent of patients have synchronous peritoneal metastases (PM) at initial diagnosis and 19% develop metachronous PM after curative primary resection, with 8% being isolated metastasis.^3,4^ Peritoneal metastases are associated with reduced overall survival, and, in 30–40% of cases, they are associated with significantly worse prognosis compared with non-peritoneal metastases.^5,6^ Contemporary systemic chemotherapy (oxaliplatin- and irinotecan-based) with targeted therapy attains median survival of 16.3 months for isolated PM, compared with 19.1 and 24.6 months for isolated liver and lung metastases.^6^

Recently, in selected patients, the combination of extensive cytoreductive surgery (CRS) with Hyperthermic intra-operative IntraPeritoneal Chemotherapy (HIPEC) has been reported to have a survival benefit for patients with peritoneal metastasis from colorectal cancer.^7-9^ CRS aims to remove all macroscopic peritoneal disease; this is achieved by resection of tumour-coated viscera and parietal peritoneum (peritonectomy). HIPEC targets the residual microscopic lesions - intraperitoneal administration allows delivery of a higher concentration of chemotherapeutics to peritoneal disease with less systemic toxicity, while mild hyperthermia enhances the drugs’ cytotoxicity and penetration into tumour nodules.^10^ CRS plus HIPEC was first used to treat appendiceal mucinous neoplasm with pseudomyxoma peritonei. This technique also proved valuable for a subgroup of colorectal PM patients. In several retrospective studies, median overall survival with CRS plus HIPEC was encouraging in patients with macroscopically complete resection.^11-13^ A recent meta-analysis showed that the treatment CRS plus HIPEC gives a significant survival advantage in carefully selected patients. The median overall survival was 30–63 months for colorectal PM.^14^ In a Dutch phase 3 controlled trial CRS plus HIPEC was superior to systemic chemotherapy in terms of overall survival in patients in whom surgery was done only to relieve symptoms caused by bowel obstruction.^8^ In specialised centres, CRS plus HIPEC can cure (*i.e*., no evidence of disease at 5 years) around 16% of patients in whom resection is macroscopically complete.^15^

Nevertheless, controversies exist on this treatment modality. Firstly, according to some studies, it carries significant morbidity and mortality; major complications and death occurred in up to 52% and 5.8% patients in high-volume centres.^14,16^ Also, the value of HIPEC on top of CRS has been questioned for colorectal PM. The results of PRODIGE-7 trial showed that combining oxaliplatin-based HIPEC with CRS had no survival advantage over optimal CRS alone, but was associated with more late complications. It is worth mentioning that both arms of PRODIGE-7 had an unexpectedly long median survival of 41 months, highlighting the benefit of complete surgical cytoreduction.^17^ Current major guidelines in Europe and the United States recommend complete CRS plus HIPEC performed in experienced centres as an appropriate treatment option for selected patients with limited colorectal PM.^18^

We have started performing CRS plus HIPEC for patients with different peritoneal tumours since 2009. To our knowledge, we are the only centre in our country performing this procedure. During this time, we have performed over 100 procedures which were performed by 5 surgeons. The aim of this study is to evaluate the efficacy and safety of CRS plus HIPEC for colorectal PM in our institute.

## Patients and methods

### Patient selection

This study is a retrospective analysis of prospectively maintained data from patients that underwent CRS followed by HIPEC for resectable PM from colorectal cancer between 2009 and 2024. The study was approved by both Institutional Ethics and Research Boards.

All patients underwent detailed preoperative assessment including radiological and/or laparoscopic staging to estimate the extent of peritoneal dissemination and resectability of the disease. Radiological staging included thoracic, abdominal and pelvic computed tomography (CT) with intravenous contrast, and/or abdominal magnetic resonance imaging (MRI). All cases were discussed in a dedicated Multi-Disciplinary Team meeting, in which treatment options were discussed. Extra-abdominal metastases or extensive small bowel/mesentery involvement were considered as absolute contra-indications to CRS plus HIPEC. Presence of synchronous liver metastases and a peritoneal cancer index (PCI) > 20 (either from imaging or exploratory laparotomy) were relative contraindications as they were associated with poor outcomes; under such circumstances, we only offer CRS plus HIPEC to young, fit patients who lack alternative treatment option (*e.g*. further systemic therapy is expected to be ineffective).

### Procedure details

In all cases, a laparotomy was performed and the extent of peritoneal disease was calculated intraoperatively using the PCI score (a scoring system that ranges from 0 to 39, and quantifies the extent of peritoneal disease based on tumour sizes and their distribution along 13 regions within the peritoneal cavity as described by Sugarbaker *et al*.).^10^ If the disease was considered to be resectable, cytoreduction was performed using tumour removal, organ resections and peritonectomy techniques as described by Sugarbaker *et al*.^10^ After the surgical procedure, the completeness of cytoreduction (CC) was evaluated for each patient as follows: a CC-0 score indicated no visible tumour in the peritoneal cavity; a CC-1 score indicated residual tumour size r < 2.5 mm; a CC-2 score indicated residual tumour size 2.5 mm–2.5 cm; a CC-3 score indicated a residual tumour size > 2.5 cm.^10^ Patients with CC-0/CC-1 scores were considered to have undergone complete cytoreduction. Following cytoreduction, patients underwent HIPEC using the closed abdomen technique. All intestinal reconstructions were performed before closure of the abdomen, six tubes (three for inflow of the chemotherapy solution and three for outflow) were inserted and the abdomen was closed with standard abdominal closure techniques. After testing for possible leaks with normal saline 0.9%, oxaliplatin was administrated in the abdominal cavity at an intraperitoneal temperature of 42 °C at a dose 360 mg/m2 for 90 minutes.

### Parameters evaluated

For each patient, demographic data, details of the course of the disease (site of primary tumour, preoperative or postoperative chemotherapy, time of diagnosis of PM, prior surgery score (PSS): PSS-0 means no prior surgery or only biopsy was made; PSS-1 limited surgery in one abdominal region; PSS-2 surgeries in two to five abdominal regions; PSS-3 extensive prior surgery > 5 regions^10^ and procedural details (PCI score, CC score) were recorded. As far as the postoperative course was concerned, the length of hospital stay was recorded, while postoperative morbidity was graded according to the Clavien-Dindo classification system.^19^ Overall survival was defined from the time of the surgical procedure to the date of reported death, and disease-free survival was defined from the time of the surgical procedure to the time of diagnosis of disease recurrence or progression.

### Statistical analysis

Overall survival was used as the primary endpoint of this study. For categorical variables, the chi-square and Fisher’s exact test were used as appropriate. Survival analysis was performed using the Kaplan–Meier method, and compared using the log-rank test. Multivariate analyses using Cox-regression models were performed in order to identify independent prognostic factors of survival. A p-value of less than 0.05 was considered statistically significant and the analysis was performed using SPSS v 20 for Windows.

## Results

Fifty patients (28 males, 22 females) with a mean age of 54 years (range 28–73 years) were included in the analysis. 22 (44%) patients had synchronous PM and 28 (56%) patients developed PM metachronous to their primary tumour. 46 (92%) patients received chemotherapy (preoperatively or postoperatively, or both). The mean intraoperative PCI score was 8.33 (range 1–35). Complete cytoreduction (CC-0/1) was achieved in 44 (88%) procedures. The median hospital stay was 15 days (range 6-114 days). Severe complications (Clavien-Dindo III/IV) were encountered in 18% of the cases whereas the 30-day mortality rate was 0%. Patient and tumour characteristics are listed in Table 1.

**TABLE 1.** Patients and tumor characteristics

	N (%)
Sex	
Male	28 (56)
Female	22 (44)
Mean age, years (range)	54 (28-73)
Diagnosis of peritoneal metastases (PM)	
Synchronous to primary tumour	22 (44)
Metachronous to primary tumour	28 (56)
Systemic chemotherapy	
No chemotherapy	4 (8)
Preoperative	28 (56)
Postoperative	35 (70)
Both	17 (34)
Peritoneal Cancer Index (PCI)	
Mean (range)	8.33 (1–35)
0–8	31 (62)
9–12	9 (18)
13–39	10 (20)
Complete cytoreduction (CC-0/1)	44 (88)
Prior surgery score (PSS)	
0	33 (66)
1	6 (12)
2	9 (18)
3	2 (4)
Median duration of hospital stay, days (range)	15 (6–114)
Clavien-Dindo III/IV	9 (18)

**FIGURE 1.**
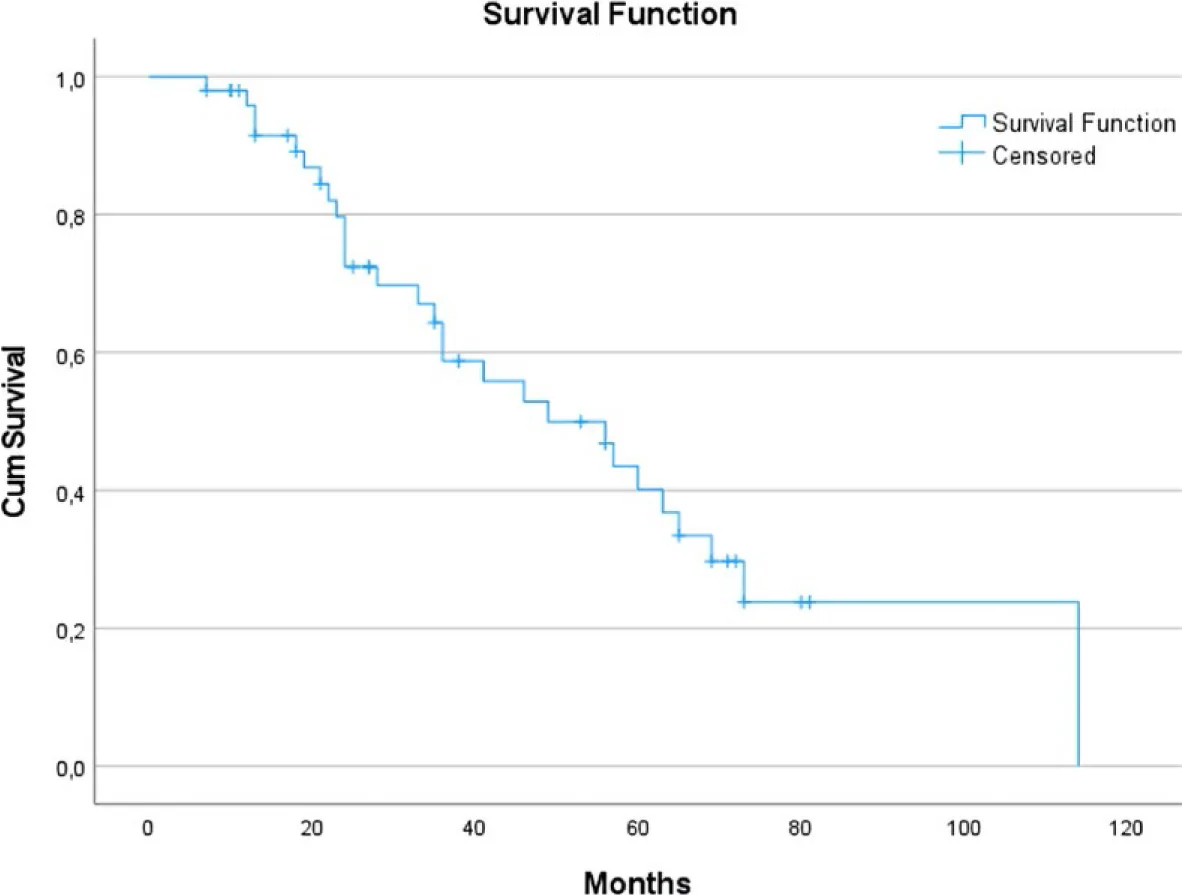
Kaplan-Meier curve: overall survival in our patient sample.

After a median follow-up of 34 months (range 7–114 months), 28 (56%) patients died of the disease. Median overall survival was 49 months. 1-year and 5-year overall survival rates were 96% and 54% (Figure 1). 33 (75%) of the 44 patients who had complete cytoreduction experienced recurrence. Median disease-free survival was 14 months. Disease-free survival rates at 1 year was 60% whereas the 5-year disease-free survival was 14.5%.

Univariate analysis showed that the PCI score, the completeness of cytoreduction (CC score) and PSS score were correlated significantly with overall survival (Table 2).

**TABLE 2.** Univariate analysis, parameters affecting overall survival

	P
Sex	Not significant (NS)
Age	NS
Diagnosis of peritoneal metastases (PM)	NS
Systemic chemotherapy	NS
Peritoneal Cancer Index (PCI) score	0.001
Completeness of cytoreduction	0.042
Prior surgery score (PSS) score	0.006

**FIGURE 2.**
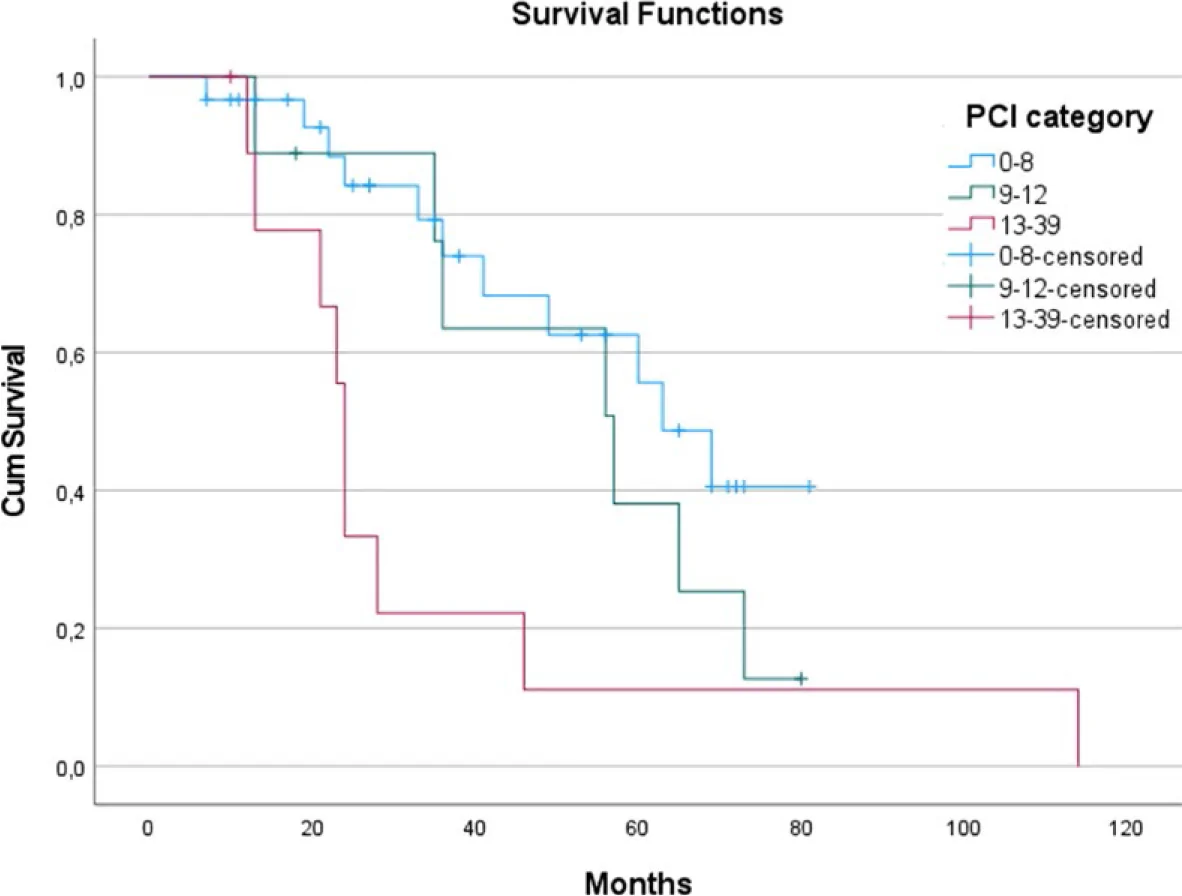
Kaplan-Meier curves: overall survival according to Peritoneal Cancer Index (PCI) score category.+

Kaplan–Meier curve analysis showed that, when the PCI was stratified in three groups (0–8, 9–2 and 13–39), there was a statistically significant difference in survival between the three groups (p = 0.014) (Figure 2, Table 3). Notably, all patients in the 0–8 group had complete cytoreduction (CC-0/1), whereas only 60% of the patients in the 13–39 PCI group were scored as CC-0/1. Statistical analysis showed a significant correlation between the PCI score and the completeness of cytoreduction (p = 0.002).

**TABLE 3.** Overall survival (OS) analysis according to Peritoneal Cancer Index (PCI) category

PCI score category	Number of patients	OS (median)	p
0–8	31	63	0.014
9–12	9	57	
13–39	10	24	

When entered in a multivariate regression model, the PCI score (p = 0.009) and the CC score (p = 0.031) were identified as independent prognostic factors of survival, adjusted for age, sex, pre/postoperative KT, time of diagnosis of PM (synchronous/metachronous) and PSS score (Table 4).

**TABLE 4.** Multivariate analysis, independent prognostic factors of overall survival

	P
Sex	Not significant (NS)
Age	NS
Diagnosis of peritoneal metastases (PM)	NS
Systemic chemotherapy	NS
Peritoneal Cancer Index (PCI) score	0.009
Completeness of cytoreduction	0.031
Prior surgery score (PSS) score	NS

## Discussion

In the past peritoneal metastasis have been considered as a terminal incurable disease. The introduction of CRS, which consists of removal of all macroscopic disease, with the addition of HIPEC in order to eradicate any residual tumour burden, has changed the management of these patients, with significant survival benefit.^20^ In selected cases of patients with colorectal PM, a median survival of up to 63 months has been reported.^7,14,21,22,23^ In the present study, we report a median overall survival of 49 months, which is in line with OS previously reported in other studies, and supports the effectiveness of the technique.

While optimal cytoreduction is universally accepted for this treatment modality, there is still a debate about the value of HIPEC on top of CRS, largely due to the heterogeneity of drugs and protocols used in HIPEC administration, plus lack of randomized trial data in the era of modern chemotherapy and targeted therapy.^24^ Since a recent randomized trial showed addition of HIPEC to CRS offered survival advantage in stage III ovarian cancer^25^ the negative finding of PRODIGE-7 trial was unexpected. One of the main criticisms of PRODIGE-7 trial was that the oxaliplatin based HIPEC was administered for only 30 min. In other protocols as well as in ours, HIPEC was administered for up to 120 min.^26^ Other studies have shown that response to local oxaliplatin is related to duration of exposure.^27,28^ Future studies of both the dose and duration of oxaliplatin-based HIPEC might produce different results. Other possible explanations for negative results of Prodige-7 trial include inappropriate choice of drug (oxaliplatin has uncertain efficacy when administered intraperitoneally) and problems in trial design (HIPEC was hypothesized to improve median OS by 18 months, on top of complete CRS; this overestimation of effect size might have obscured any potential subtle benefits of HIPEC).^29^ More research efforts should be directed at identifying the optimal drug and protocol for HIPEC for colorectal PM and establishing criteria to select the subgroup that will benefit most from HIPEC.^24^ Currently most Western guidelines recommend CRS plus HIPEC as a standard therapy for selected colorectal PM patients.^18,24^

During the last decade, a lot of studies have investigated prognostic factors of outcome in patients undergoing CRS and HIPEC, in order to optimize selection of patients who will benefit more from this aggressive surgical approach.

Completeness of cytoreduction (CC-0/1) has been shown to be the most important prognostic factor, as 5-year survival in patients undergoing complete cytoreduction has been reported to be higher compared to that of patients undergoing incomplete resections.^22^ In our study, patients with CC score of 0 or 1 enjoyed significantly better OS. Therefore, if incomplete cytoreduction is likely, CRS plus HIPEC should not be carried out.

The extent of peritoneal disease based on tumour sizes and their distribution along 13 regions within the peritoneal cavity (PCI score) remains another significant prognostic factor. However, the specific cut-off point associated with poor prognosis has yet to be defined, with the limit currently being between 10 and 20.^18,30^ Sugarbaker *et al*. initially reported significantly higher survival (41 months) in patients with a PCI score less than 20, compared to 16 months for patients with a PCI score greater than 20.^31^ Yan and Morris later showed that a PCI score ≤ 10 was a significant favourable prognostic factor.^32^ In the present study, our analysis confirms that the PCI score remains a significant prognostic indicator of survival. There was a significant difference in the survival of patients according to the peritoneal tumour burden (PCI scores 0–8, 9–12 and 13–39), and PCI score of 13 or more was associated more frequently with incomplete resection.

High morbidity and mortality reported in some studies have discouraged many surgeons and oncologists from referring suitable patients for CRS plus HIPEC. With technological advances, these rates have improved to 22–34% and 0–4.1% in recent large series.^14,33^ An analysis of the US national surgical database showed CRS plus HIPEC was a safer procedure than Whipple operation and esophagectomy.^34^ Our results were similar, with a grade III or above complication rate of 18% and zero 30-day mortality.

Our study shows that CRS plus HIPEC offers survival benefit to patients with colorectal PM with small to moderate volume PM, with reasonable morbidity and mortality. Appropriate patient selection is the key to achieving good outcome, and complete cytoreduction is an important prognostic factor.
